# Supplementary material for: A Model Construction of Starvation Induces Hepatic Steatosis and Transcriptome Analysis in Zebrafish Larvae
Source: Biology (Basel). 2021 Jan 27;10(2):92. doi: 10.3390/biology10020092 (PMC7911188; doi:10.3390/biology10020092)
Supplement: Supplementary file 1 [file biology-10-00092-s001.zip › Supplementary files/Table S1.docx]

| Gene | Primer sequence (5'-3') | Usage |
| --- | --- | --- |
| *cd36*-Cas-F | TAATACGACTCACTATAGGCGGAATCCTCATCCCGGTGTTTTAGAGCTAGAAATAGC | Gene knockout |
| *oxr1a*-Cas-R | aaaaaaagcaccgactcggt |  |
| *cd36_exon1_*-F | AACTCCGTCCTAACGAGGGT | Mutation analyses |
| *cd36_exon1_*-R | tccttgacccaaaaacccca |  |
| *cd36*-PAGE-F | ATCAGCGGTGCGCACTCATCA | Mutation analyses |
| *cd36*-PAGE-R | GACGACGACTTACCTTATGG |  |
| Q-*cpt1ab*-F | atgagagcaaacacatcgcc | Real-time PCR |
| Q-*cpt1ab*-R | agcagccaaaaactcttcacc |  |
| Q-*cpt1b*-F | TGGCACTGCAACTAGCTCAA | Real-time PCR |
| Q-*cpt1b*-R | CGTGGTTGTGTCATCCTCCA |  |
| Q-*cpt2*-F | agcagtaaggatggagcagga | Real-time PCR |
| Q-*cpt2*-R | attgtattcgggtttggggt |  |
| Q-*acox1*-F | CAAGAGCCCCTAGGTGTCCA | Real-time PCR |
| Q-*acox1*-R | GCAGTGGTTTCAAGAGCACG |  |
| Q-*acaa1*-F | ATGCAATCCCAGAAGCCCTC | Real-time PCR |
| Q-*acaa1*-R | CACCACGCCAAATCCTCTCT |  |
| Q-*hadhab*-F | AGATATTGCAGCCGTGAGCA | Real-time PCR |
| Q-*hadhab*-F | ATATACGCACTGCCTCTGCC |  |
| Q-*dgat1a*-F | CCAAAGCTCGAACCCTGTCT | Real-time PCR |
| Q-*dgat1a*-R | TCTGTGTGTGAGGTTTCCCG |  |
| Q-*dgat1b*-F | GAGTTGCCACAAGTTGCAGG | Real-time PCR |
| Q-*dgat1b*-R | TGCAGCAGGCCAGCTATAAG |  |
| Q-*dgat2*-F | GGCATTGCTTGCTCTTTGCT | Real-time PCR |
| Q-*dgat2*-R | ACAAGATGCCATGAGGGTGG |  |
| Q-*srebf1*-F | AGGGAGAGCATCTACAGCCA | Real-time PCR |
| Q-*srebf1*-F | ATGGCGAAGGATTGTGTCGT |  |
| Q-*srebf2*-F | CTCTAAGCCCCTCCCAGACT | Real-time PCR |
| Q-*srebf2*-F | GGGGTCCGCTTTATCTCTCG |  |
| Q-*fasn*-F | ACGGCAATGTCACCCTACTG | Real-time PCR |
| Q-*fasn*-R | ATGCGAAGGTTTAGCCCTCT |  |
| Q-*scdb*-F | GAAGCAATGGCCGATGTGAC | Real-time PCR |
| Q-*scdb*-R | GCGCTCCTAGGTGCAATAGA |  |
| Q-*apoa1b*-F | GATGAGGTTCGTAGCCCTCG | Real-time PCR |
| Q-*apoa1b*-R | TGAGTGAGGGACTTGTGTGC |  |
| Q-*apoa4b.3*-F | GAGAAGCTGGACCCCTATGC | Real-time PCR |
| Q-*apoa4b.3*-R | TGGCATGATAGGATGAATCGATAG |  |
| Q-*apoba*-F | CTCTTTGGAGAGCGCTTGGA | Real-time PCR |
| Q-*apoba*-R | AGCGTGGAACGAAGACCATT |  |
| Q-*slc27a2a*-F | CGTGCTTCTCCACACTCGAT | Real-time PCR |
| Q-*slc27a2a*-R | TGCATCCCGGTAAGTGTAGC |  |
| Q-*fabp10a*-F | AACTCCTTCACCATCGGCAA | Real-time PCR |
| Q-*fabp10a*-R | ATCATGGTGGTTCCTCCGAC |  |
| Q-*fabp1b.1*-F | GGAGAGTCAAGAGGGCTTCG | Real-time PCR |
| Q-*fabp1b.1*-R | GCACTTTTGATCCGGTCGTG |  |
| Q-*cd36*-F | GGTCGGAATGAACCCCAACT | Real-time PCR |
| Q-*cd36*-R | TGTCCATCCGGGAAATCAGC |  |
| Q-*eef1a1l1*-F | gcttctctacctaccctcctct | Reference gene |
| Q-*eef1a1l1*-R | caccaccgattttcttctca |  |

**Table S1. Sequences of primers used in this study**
