# Supplementary material for: A Model Construction of Starvation Induces Hepatic Steatosis and Transcriptome Analysis in Zebrafish Larvae
Source: Biology (Basel). 2021 Jan 27;10(2):92. doi: 10.3390/biology10020092 (PMC7911188; doi:10.3390/biology10020092)
Supplement: Supplementary file 1 [file biology-10-00092-s001.zip › Supplementary files/Table S2.docx]

Table S2 Genes expressed only in the control and starved larvae

| Genes expressed only in the control larvae | Gene_id | Genes expressed only in the starved larvae | Gene_id |
| --- | --- | --- | --- |
| *FO904901.1* | ENSDARG00000106745 | *endouc* | ENSDARG00000112234 |
| *si:dkey-83f18.2* | ENSDARG00000055346 | *dpm2* | ENSDARG00000095623 |
| *opn1mw1* | ENSDARG00000097008 | *ywhag2* | ENSDARG00000071658 |
| *FP085399.5* | ENSDARG00000104882 | *larp6b* | ENSDARG00000003968 |
| *galm* | ENSDARG00000057630 | *admb* | ENSDARG00000069027 |
| *faap24* | ENSDARG00000051731 | *olfm3b* | ENSDARG00000039174 |
| *zgc:194906* | ENSDARG00000095773 | *asmt* | ENSDARG00000098249 |
| *si:dkey-61p9.7* | ENSDARG00000068865 | *dlgap2b* | ENSDARG00000076070 |
| *eri2* | ENSDARG00000104392 | *tmem119b* | ENSDARG00000068036 |
| *thbd* | ENSDARG00000092470 |  |  |
| *znf1023* | ENSDARG00000086720 |  |  |
| *BX950187.1* | ENSDARG00000095282 |  |  |
| *tnfsf13b* | ENSDARG00000012945 |  |  |
| *si:ch73-269m14.4* | ENSDARG00000086612 |  |  |
| *spink2.5* | ENSDARG00000101290 |  |  |
| *BX323064.2* | ENSDARG00000104365 |  |  |
| *klhdc4* | ENSDARG00000100206 |  |  |
| *thoc6* | ENSDARG00000037966 |  |  |
| *si:ch73-338o16.4* | ENSDARG00000115257 |  |  |
| *gpa33b* | ENSDARG00000040898 |  |  |
| *dctpp1* | ENSDARG00000054334 |  |  |
| *BX001030.1* | ENSDARG00000093671 |  |  |
| *pex11a* | ENSDARG00000060707 |  |  |
| *ifit15* | ENSDARG00000043584 |  |  |
| *CU469568.2* | ENSDARG00000094923 |  |  |
| *dusp23a* | ENSDARG00000009844 |  |  |
| *LO017725.1* | ENSDARG00000117204 |  |  |
| *AL845330.1* | ENSDARG00000108467 |  |  |
