# Supplementary figures and images for: A Model Construction of Starvation Induces Hepatic Steatosis and Transcriptome Analysis in Zebrafish Larvae
Source: Biology (Basel). 2021 Jan 27;10(2):92. doi: 10.3390/biology10020092 (PMC7911188; doi:10.3390/biology10020092)

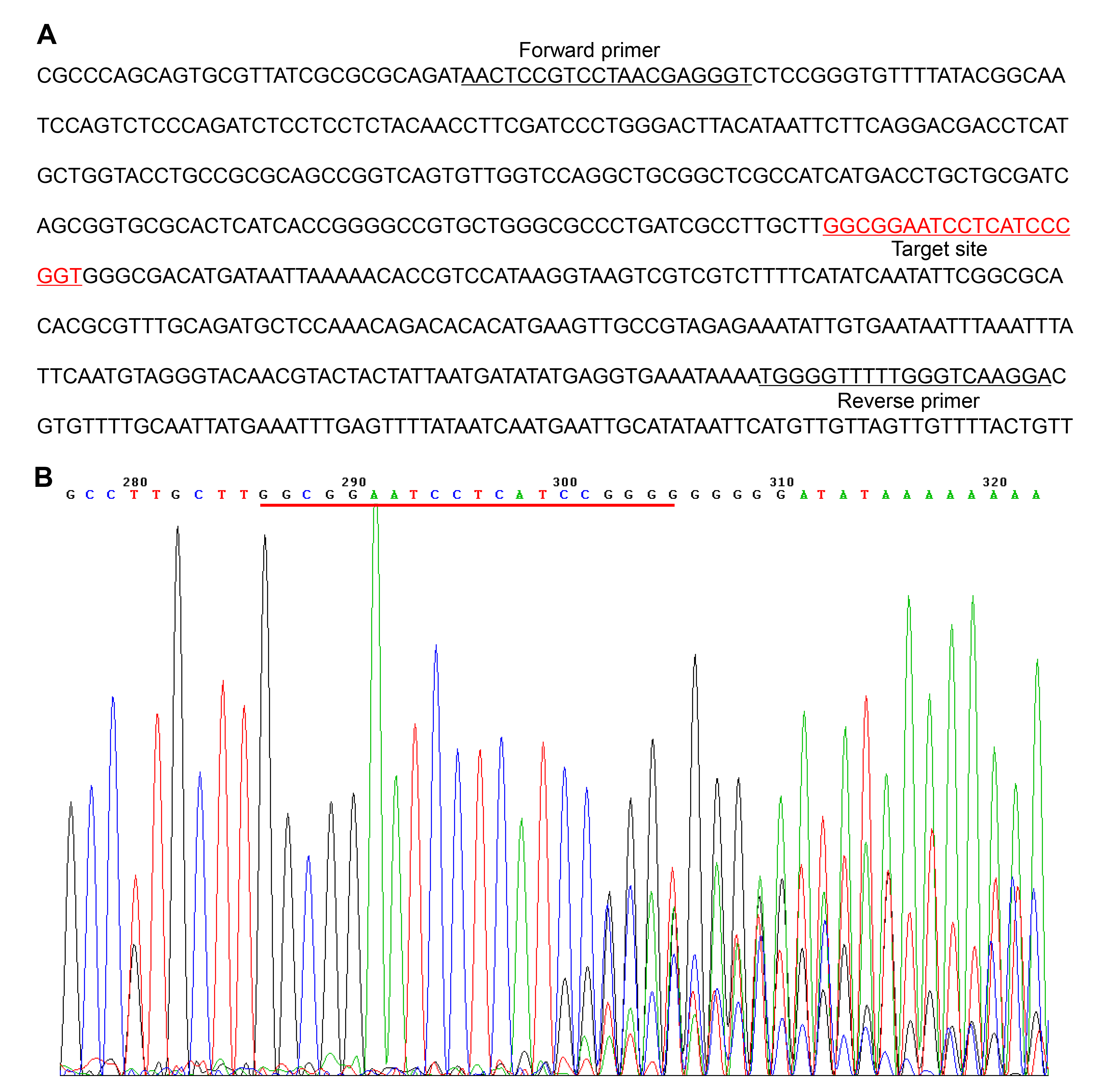

Supplement: Supplementary file 1 [file biology-10-00092-s001.zip › Supplementary files/Figure S1.tif]
